# Efficient Acyloxymethylation of Psilocin and Other Tryptamines Yielding ACOM Prodrugs for Psychedelic‐Assisted Therapy

**DOI:** 10.1002/ardp.70022

**Published:** 2025-07-23

**Authors:** Judith Stirn, Christian D. Klein

**Affiliations:** ^1^ Medicinal Chemistry, Institute of Pharmacy and Molecular Biotechnology IPMB Heidelberg University Heidelberg Germany

**Keywords:** ACOM prodrug, buccal/sublingual, psilocin, psychedelic‐assisted therapy, tryptamine

## Abstract

Acyloxymethyl (ACOM) derivatives of tryptamines such as the psychedelic drug psilocin and the anti‐migraine drug sumatriptan bear potential as prodrugs. Previous synthetic approaches suffer from insufficient chemoselectivity between the desired functionalization of the phenolic (psilocin) or sulfonamide (sumatriptan) groups versus other reactive groups in the parent drugs. We report a novel synthetic route toward ACOM prodrugs of tryptamines via the chemoselective installation of a carbamate protecting group at the indole nitrogen by means of a Heller–Sarpong reagent and final deprotection under extremely mild conditions. This enables delicate transformations such as the *O*‐acyloxymethylation of psilocin or the *N* ^*2*^‐acyloxymethylation of sumatriptan. Several novel *O*‐ACOM ethers of hydroxytryptamines were obtained and evaluated in vitro for their potential as novel prodrugs for psychedelic therapy. The rate of bioactivation in human plasma may be adjusted to rapid (*t*_1/2_ < 1 min) or slow (*t*_1/2_ > 240 min) kinetics by varying the acyl residue in the ACOM promoiety. Irrespective of the acyl residue, short half‐lives in human saliva will likely preclude the sublingual or buccal application of ACOM ether prodrugs of hydroxytryptamines, while other routes such as peroral, transdermal, nasal, or intravenous administration may be pursued.

## Graphical abstract

Chemoselective *N* ^*1*^‐protection of multifunctional indoles by means of a Heller–Sarpong reagent, followed by acyloxymethylation and mild hydrogenolytic deprotection, affords novel acyloxymethyl (ACOM) derivatives. Several ACOM ethers of 4‐hydroxytryptamines such as psilocin were prepared and characterized in vitro, revealing a tailorable bioactivation rate in human plasma and an unanticipated lability in human saliva. Administered via the peroral, transdermal, nasal, or intravenous route, these prodrugs may open new avenues for psychedelic therapy.

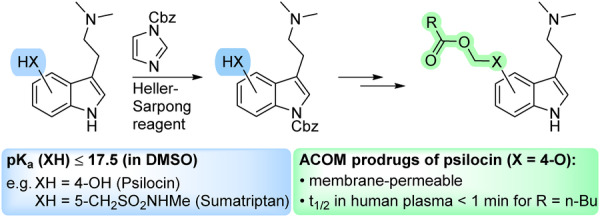

## 1 Introduction

Tryptamines such as psilocin **1** and the anti‐migraine triptans are of considerable interest in medicinal chemistry. Psilocin, a 5‐HT_2A_ agonist, is currently under evaluation in multiple clinical trials for the treatment of depressive disorders (e.g., NCT04670081, NCT05624268), where it is dosed perorally in the form of its highly polar phosphate ester prodrug psilocybin [1]. Sumatriptan **3**, a representative of sulfonamide‐bearing triptans, is an anti‐migraine drug of high clinical significance [2] and exerts its therapeutic effects on cerebral blood vessels and sensory nerves of the trigeminal system by activation of 5‐HT_1B/1D_ subtype receptors [3]. Both psilocybin and sumatriptan display pharmacokinetic shortcomings such as high polarity (logD_7.4_(psilocybin) = −0.89, logD_7.4_(sumatriptan) = −1.3) [4], first‐pass metabolism ascribed to monoamine oxidase [5], and substantial interindividual variability of plasma concentrations after peroral administration [6].

Administration routes beyond the classical peroral or intravenous mode may be used to implement an optimized therapy, as exemplified by zolmitriptan nasal sprays for the acute treatment of migraine [7]. However, intranasal, buccal, sublingual, and transdermal administration require efficient permeation of the drug across biological membranes. Therefore, tryptamine prodrugs with enhanced lipophilicity and thus improved passive membrane permeation profile are of great interest for therapeutic applications. The lipophilic promoiety of such prodrugs may be further tailored to obtain desirable release kinetics and increased shelf life, thus providing additional benefits compared to the parent drug [8].

Recently, acyloxymethyl (ACOM) derivatives of tryptamines have been pursued for this purpose. These include *N* ^*1*^‐ACOM derivatives of sumatriptan [9] and psilocin [10] and *O*‐ACOM derivatives of psilocin [10, 11] and psilocybin [10b]. *N* ^*1*^‐ACOM derivatives of tryptamines are synthetically easier to access than their *O*‐regioisomers. However, their hydrolytic activation generates relatively stable *N* ^*1*^‐hydroxymethyl intermediates, which significantly delays or impedes the release of the parent drug as observed experimentally [9, 12]. Under physiological conditions (pH 7.4, 37°C), a half‐life of nearly 40 days is predicted for *N* ^*1*^‐hydroxymethyl indoles by the structure–reactivity relationship [13] established by Bundgaard and Johansen (p*K*_a_(indole)~17 in water) [14]. This would not be compatible with the fast release required for psychedelic treatment sessions. Few other approaches to installing a promoiety at the indole nitrogen have been tested [12, 15], but the indole nitrogen appears to be generally of limited use as a handle for prodrug design. Hence, *N* ^*1*^‐protection with a promoiety is not a viable approach toward ACOM prodrugs of indole‐derived drugs, such as the tryptamines.

Consequently, there is a need for synthetic access to *N* ^*1*^‐unsubstitued ACOM derivatives of drugs featuring an indole scaffold to obtain prodrugs with therapeutically useful release kinetics. Given the relatively low p*K*_a_ value of phenols and the expected rapid release of the hemiacetal that is formed in situ, aromatic hydroxy groups of tryptamines like psilocin are particularly attractive handles for installing ACOM promoieties.

However, *O*‐ACOM prodrugs of psilocin **1** and other 4‐hydroxytryptamines are difficult to access synthetically due to (1) the presence of three (pro)nucleophilic sites in psilocin (indole nitrogen, aliphatic amino group, and phenolic hydroxy group); (2) the presence of two electrophilic sites in the commonly used halogenomethyl carboxylate reagents (ester moiety and chloromethyl moiety) [16]; and (3) the lability of the acetal linker [17]. As a consequence, the yield of such transformations is generally low, as evidenced by recently published patent applications [10].

**Figure 1:**
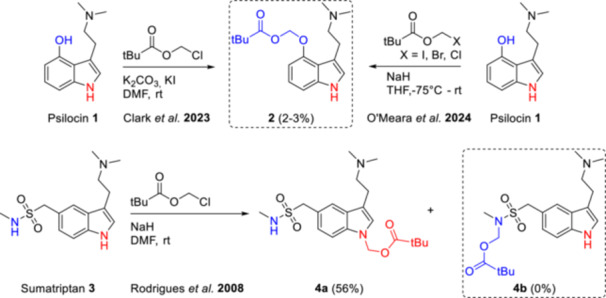
Previously reported, direct acyloxymethylation of psilocin and sumatriptan along with isolated yields. The targeted *N* ^1^‐unsubstituted derivatives (framed with a dashed line) remained elusive or were obtained in minuscule amounts [9, 10].

The first publicly available account on ACOM ethers of psilocin appeared in 2023 and reports the synthesis of only one representative, the pivaloyloxymethyl ether **2**. The targeted transformation was effected by reacting psilocin with chloromethyl pivalate in the presence of KI and K_2_CO_3_. Notably, three subsequent chromatographic purifications were required to obtain the compound in sufficient purity, and the yield was extremely low (2%, Figure 1) [10b].

Also in 2023, two additional *N* ^*1*^‐unsubstituted ACOM ethers of psilocin were reported in patent and academic literature, and many more were claimed [11]. Surprisingly, yields of 27%–54% were reported for the *n*‐butyryloxymethyl and *n*‐pentanoyloxymethyl ethers despite similar reaction conditions to the first account. However, as reported in detail below, we must assume that the authors inadvertently isolated *N*‐ACOM derivatives of psilocin and mistook them for the targeted ACOM ethers.

Only months later, in early 2024, a patent application claiming the synthesis of the *iso‐*butyryloxymethyl ether of psilocin was published [18]. Except for a superficial reaction scheme, no information regarding reaction conditions, purification procedure, yield, and analytical characterization was provided, which questions the experimental evidence for the claim. Finally, another patent application disclosed the successful synthesis of the previously reported pivaloyloxymethyl ether **2**, this time via deprotonation with NaH at cryogenic temperatures (−75°C) followed by addition of the haloalkane. The targeted product was isolated in a marginally improved yield of 3% [10a].

For sumatriptan, previous attempts toward the acyloxymethylation of the sulfonamide side chain failed entirely, likely due to a lack of chemoselectivity [9].

In the present work, we address the challenging synthesis of *N* ^*1*^‐unsubstitued ACOM derivatives of drugs featuring an indole scaffold using 4‐hydroxytryptamines as model drugs. We proceed by extending our method to sumatriptan and finally discuss conceivable routes of administration for ACOM ethers of psilocin based on their stability profile in human blood plasma and saliva.

## 2 Results and Discussion

### 2.1 Chemistry

**Figure 2:**
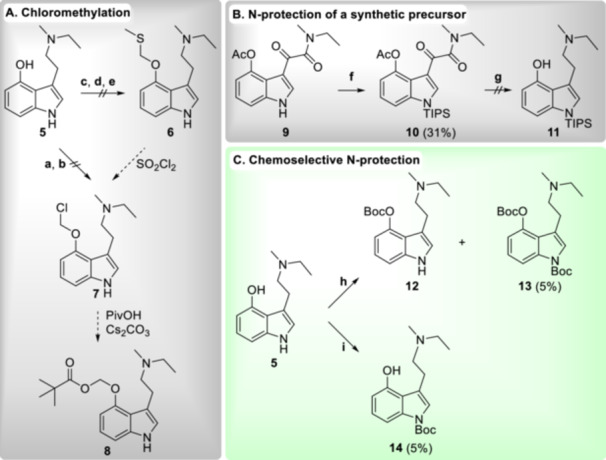
Discarded (gray boxes) and successful (green box) approaches to an improved acyloxymethylation procedure. Reaction conditions: **a.** Ar, THF, 60°C–65°C 1) 2.00 eq NaOH, 1 h; 2) 30.0–90.0 eq CH_2_BrCl, 2–3 h; **b.** DME 1) 1.05 eq NaH, 0°C, 15 min; 2) 5.00 eq ICH_2_Cl, 0°C – rt, 20 h; 3) 80°C, 3.5 h; **c.** 1.10 eq Ag_2_O, 1.10 eq MeSCH_2_Cl, Ar, MeCN, rt, 17 h; **d.** DMF 1) 1.20 eq NaH, 0°C, 30 min; 2) 1.20 eq MeSCH_2_Cl, 0°C–rt, 12 h; **e.** 1.10 eq AgOTf, 1.10 eq MeSCH_2_Cl, Ar, MeCN, rt, 2 h; **f.** THF 1) 1.10 eq LiHMDS, 0°C, 15 min; 2) 1.20 eq TIPSCl, 0°C – rt, 2 h; **g.** 3.20 eq LiAlH_4_, 2‐Me‐THF, 80°C, 4–40 h; **h.** 0.20 eq DMAP, 1.02 eq Boc_2_O, DCM/NEt_3_ (11:1), rt, 3 h; **i**. 1.10 eq. 1‐Boc‐imidazole, 0.50 eq DBU, MeCN, rt, 24 h.

With respect to a comparative evaluation of various prodrugs, the modular assembly of the ACOM promoiety would be very attractive. Such a strategy has already proven advantageous for the synthesis of a library comprising ACOM ether fluorescein derivatives, which sparked our interest in the *O*‐chloromethyl ether **7** as versatile synthetic intermediate (Figure 2A) [19].

Despite multiple synthetic attempts and experimental variations, compound **7** remained elusive in this approach, presumably due to chemical instability and/or a lack of chemoselectivity. In addition, the reaction of **5** with iodomethyl pivalate failed (not shown), which is in line with the tedious purification and minuscule yield reported for the closely related pivaloyloxymethyl ether of psilocin **2** (2%–3%) [10].

Aiming to reduce the number of (pro)nucleophilic sites and thus potential side reactions, we devised a concise protecting group strategy for the *O*‐acyloxymethylation of psilocin and related tryptamines. *N^1^*‐protection of a pre‐differentiated synthetic intermediate such as the glyoxylamide **9** or 4‐benzyloxy‐*N*‐ethyl‐*N*‐methyltryptamine appeared straightforward at first glance but was found to have several limitations: (1) The choice of protecting groups is restricted to relatively stable ones that are compatible with reductive reaction conditions (LiAlH_4_ or H_2_, Pd/C). Such protecting groups require deprotection conditions that are potentially too harsh and likely not orthogonal to the acyloxymethyl promoiety (labile toward acids, bases, nucleophiles, and heat); (2) reduction of *N^1^*‐protected glyoxylamides such as **10** with LiAlH_4_ is sluggish (Figure 2B), presumably because the protecting group precludes the involvement of the indole nitrogen in the rate‐limiting reduction of the β‐hydroxy intermediate [20]; (3) *O*‐benzyl intermediates are potentially unstable [21]; and (4) the atom and step economy of the *O*‐benzyl route is unsatisfactory.

To our delight, however, we found that the indole nitrogen of 4‐hydroxytryptamines can be acylated in a chemoselective manner by Heller–Sarpong reagents (1*H*‐imidazole‐1‐carboxylates). As demonstrated by Heller et al., the acyl transfer is reversible in the presence of 1,8‐diazabicyclo[5.4.0]undec‐7‐ene (DBU) and imidazole, resulting in the formation of the thermodynamically most stable, *N* ^*1*^‐substituted product [22]. We first tested this transformation with the commercially available reagent 1‐Boc‐imidazole (Figure 2C, i) and observed the slow formation of **14** (c.f. Supporting Information S1: Figures S6, S7), which we confirmed by its isolation from the not yet equilibrated reaction mixture after a reaction time of 24 h. In contrast to this, reaction with di‐*tert*‐butyl dicarbonate was governed by kinetic control and favored *O*‐protection (Figure 2C, h). In both cases, the low isolated yields are attributable to incomplete consumption of the starting material and the difficult chromatographic purification of the tryptamine species from the complex reaction mixtures.

**Figure 3:**
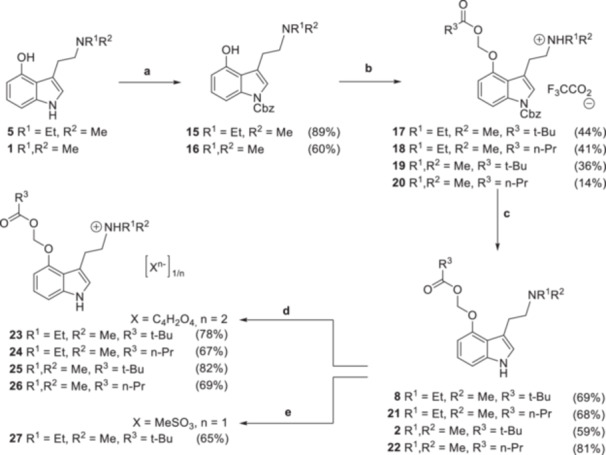
Overview of the synthetic route. Reaction conditions: **a.** 1.10 eq. 1‐Cbz‐imidazole, 0.50 eq DBU, MeCN, rt, 18–26 h; **b.** DMF/THF, −50°C to rt 1) 1.50 eq NaH, 5–30 min; 2) 1.00–1.50 eq iodomethyl carboxylate, 2–5 h; **c.** 1 atm H_2_, 12–15 mol% Pd/C, EtOAc, rt, 4.5–16 h; **d.** 0.50 eq fumaric acid, acetone, 5°C, 1.5–6 h; **e.** 1) 1.00 eq MeSO_3_H, acetone, 5°C, 2 h; 2) cyclohexane.

To ensure mild *N^1^*‐deprotection conditions compatible with the ACOM ether moiety, we next prepared the Heller–Sarpong reagent 1‐Cbz‐imidazole and reacted it with the 4‐hydroxytryptamines **1** and **5**. While equilibration using 1‐Boc‐imidazole was slow, likely due to steric hindrance, 1‐Cbz‐imidazole proved to be a suitable reagent to achieve prompt *N*‐protection under mild conditions (Figure 3).

The structural assignment of the formed carbamates **15** and **16** is based on various spectroscopic characteristics (c.f. Supporting Information S1: Figures S8, S9; Table S7). First, the ^1^H NMR spectra of **1**, **5**, **15**, and **16** have a broad singlet between 12.5 and 14.0 ppm in common, which is typical of a phenolic OH resonance. All other ^1^H NMR resonances with chemical shifts above 5.5 ppm can be clearly traced back to aromatic CH groups as evidenced by means of ^1^H,^13^C HSQC (heteronuclear single quantum correlation). Second, the chemical shift of the benzylic methylene group is indicative of *N*‐ versus *O*‐substitution. This moiety gives rise to a singlet at 5.42 ppm in the ^1^H NMR spectra of **15** and **16**, in line with a reported chemical shift of 5.46 ppm for the *N^1^*‐Cbz reference compound benzyl 1*H*‐indole‐1‐carboxylate [22, 23] but somewhat downfield‐shifted as compared to the *O*‐Cbz reference compound benzyl phenyl carbonate (5.23–5.27 ppm [24]). The differences in the chemical shifts are even larger in the ^13^C NMR spectra, where the benzylic methylene resonance of **15**, **16**, and benzyl 1*H*‐indole‐1‐carboxylate [23b] is observed at 68.5–68.6 ppm while 70.3 ppm is reported for benzyl phenyl carbonate [24a].

Next, the carbamates **15** and **16** were allowed to react with selected iodomethyl carboxylates. These are easily accessible from commercially available chloromethyl carboxylates by a Finkelstein reaction [25] and were obtained in excellent yield as analytically pure colorless oils after aqueous workup of the reaction mixture. Several methods were tested for purifying the crude product obtained by reacting iodomethyl carboxylates with carbamates **15** and **16**. Column chromatography using silica (EtOAc/MeOH gradient, 1% NEt_3_) and Florisil® (Cyclohexane/EtOAc gradient and EtOAc/ACN gradient) as stationary phases failed to afford the pure product. However, RP chromatography on a C‐18 column using an ACN/H_2_O (+0.1% TFA) gradient was found to yield the targeted products as trifluoroacetates. The salt form was not converted into the free base as protonation of the amino group was considered advantageous for the subsequent Pd‐catalyzed hydrogenolysis of the carbamate moiety. Indeed, no reaction occurred when the crude product obtained in the acyloxymethylation step was directly subjected to hydrogenolysis in ethyl acetate supplemented with 0.1% of the relatively weak acetic acid. To the contrary, the HPLC‐pure tryptammonium trifluoroacetates **17**–**20** did not poison the catalyst and were quantitatively deprotected within a few hours. This suggests that quantitative protonation of the tertiary amino group by a strong acid is pivotal for an efficient Pd‐catalyzed deprotection of *N*‐Cbz tryptamines. To rule out palladium contamination of the final product, the deprotected TFA salts were subjected to HPLC purification. Basic aqueous workup of the eluted product fractions yielded the free bases of the ACOM ether prodrugs **8**, **21**, and **22** as viscous oils and **2** as colorless solid. Notably, the sequence of *N*‐Cbz protection, acyloxymethylation, and hydrogenolysis (Figure 3) afforded **2** with a more than sixfold higher yield than the direct acyloxymethylation of psilocin [10b] (Figure 1), which demonstrates the utility of our synthetic methodology.

The free bases of the ACOM ether prodrugs **2**, **8**, **21**, and **22** were finally precipitated from acetone as pharmaceutically acceptable tryptammonium fumarates (2:1 stoichiometry) **23**–**26**. These were obtained as colorless, free‐flowing solids that are more suitable for handling and storage than the viscous free bases. In addition, the mesylate and hydrochloride salt forms were targeted. The mesylate **27** initially formed an oil which slowly solidified upon standing, whereas the hydrochloride remained elusive due to the decomposition of **8** upon the addition of HCl in diethyl ether.

**Figure 4:**
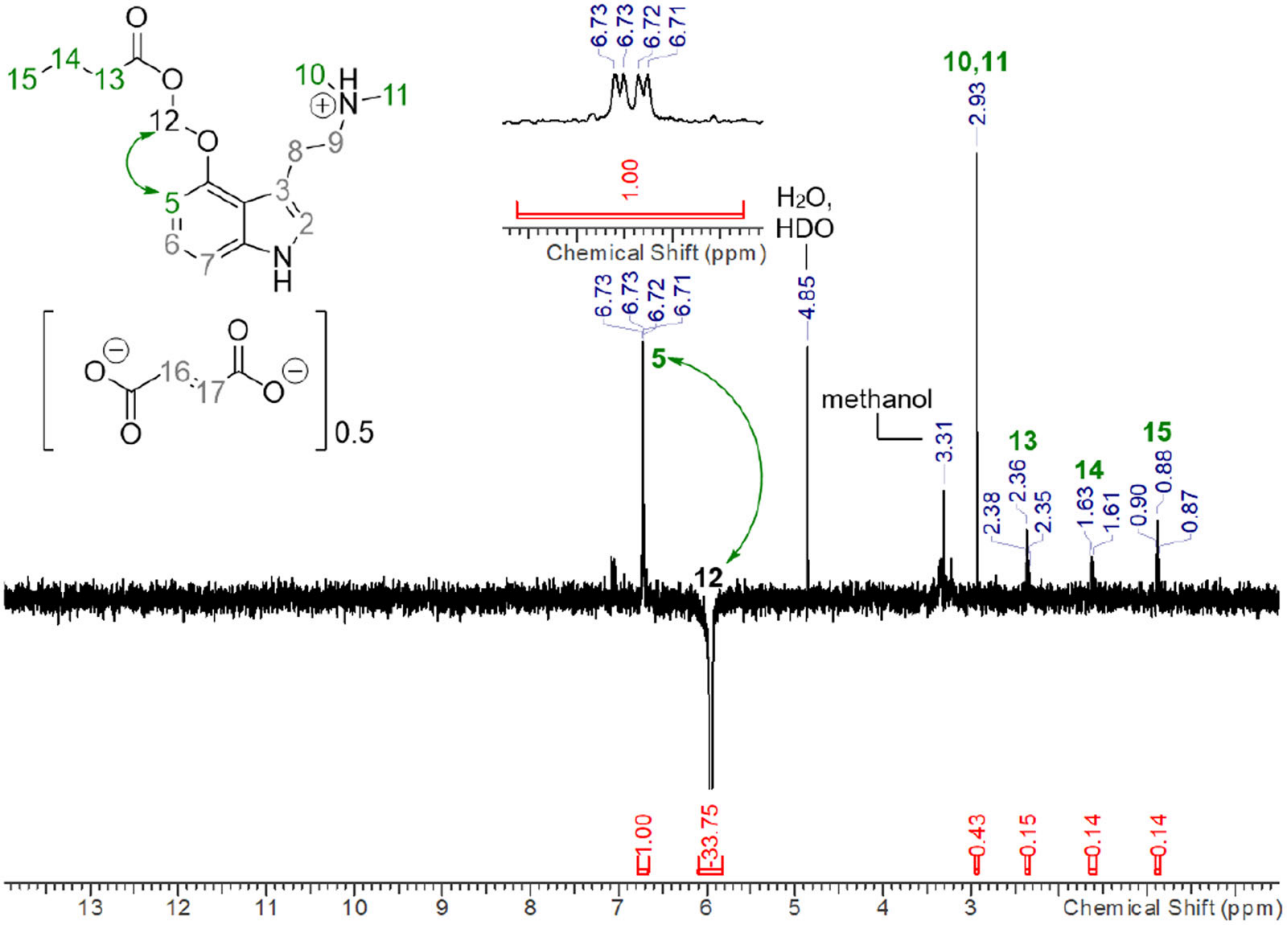
1D NOE spectrum (500 MHz, CD_3_OD, 500 ms mixing time) of compound **26** complemented with our proposed assignment. The resonance at 5.95 ppm (assigned to the methylene group of the acetal linker, 12) was selectively inverted by irradiation. As the protonated tryptamine (305 g mol^−1^) is a relatively small molecule, the nuclear spins involved in the observed NOEs are expected to be maximum 4 Å apart [27].

Of note, the spectroscopic properties of **22** and **26** contradict previous publications claiming the synthesis of **22** (“*EE01/D(IV)*”) and its pentanoyl congener (“*EE02/D(III)*”) by simply stirring a mixture of psilocin, chloromethyl butyrate/pentanoate, KI, and K_2_CO_3_ in DMF at room temperature (Table 1) [11]. Considering the presence of three (pro)nucleophilic groups in the psilocin scaffold and the identical molecular mass of **22** and *EE01/D(IV)*, we suspected them to be regioisomers. To rule out any spectral misinterpretations on our side, we acquired ^1^H; ^13^C [26]; ^1^H,^1^H COSY; ^1^H,^13^C HSQC; and ^1^H,^13^C HMBC NMR spectra of **26** in both DMSO‐d_6_ and methanol‐d_4_, complemented with a 1D NOE spectrum in methanol‐d_4_ (Figure 4).

**Table 1:** Overview of reported *N* ^*1*^‐unsubstituted ACOM ethers of psilocin and other 4‐hydroxytryptamines. One additional patent application [18] claims the isobutyryl derivative but does not provide any experimental evidence and was hence excluded.

	Valid prior reports [10]	This study^a^	Erroneous claims [11]
Scope	**2** b	**2** R^1^,R^2^ = Me; R^3^ = *t‐Bu* **8** R^1^ = Et, R^2^ = Me; R^3^ = *t‐*Bu **21** R^1^ = Et, R^2^ = Me; R^3^ = *n‐*Pr **22** R^1^,R^2^ = Me; R^3^ = *n‐*Pr And salt forms **23**‐**27**	R^1^,R^2^ = Me; R^3^ = *n*‐Pr *EE01/D(IV)* R^1^,R^2^ = Me; R^3^ = *n*‐Bu *EE02/D(III)*
Overall yield	2%–3%	7%–27%	26%–54%
MS	Conforms	Conforms	Conforms
NMR solvents	CDCl_3_	**2**, **22**: CD_3_CN, **26**: CD_3_CN, CD_3_OD	CD_3_OD
δ(N(C*H*_*3*_)_2_ (ppm)	2.90 (s, 6H) [10a] 2.38 (s, 6H) [10b]	Free base: 2.23–2.26 (s, 6H, **2**, **22**) Fumarate: 2.63 (CD_3_CN, s, 6H, **26**) 2.93 (CD_3_OD, s, 6H, **26**)	2.93 (d, *J* = 4.5 Hz, 7H, *D(IV)*) [11a] 2.91–2.93 (s, 6H, *EE01*, *EE02*, *D(III)*) [11]
δ(X‐C*H*_*2*_‐O) (ppm)	5.92 (s, 2H) [10a] 5.96 (s, 2H) [10b]	Free base: 5.86–5.87 (s, 2H, **2**, **22**) Fumarate: 5.90 (CD_3_CN, s, 2H, **26**) 5.95 (CD_3_OD, s, 2H, **26**)	5.59–5.60 (s, 2H, *EE01*, *D(IV)*, *EE02*) [11] 6.17 (s, 2H, *D(III)*) [11a]
Structures proposed by us	Free base: lit [10b] and **2**, **8**, **21**, **22** 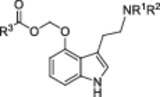		*EE01*, *EE02*, *D(IV)* 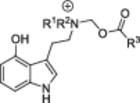
	Salt form: lit [10a] and **23**–**27** 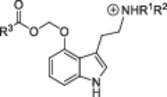		*D(III)* 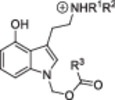

^a^ For clarity, only the spectroscopic data of **2**, **22** and **26** are listed. See Supporting Information for further information.

^b^ Both patent applications claim the free base but one [10a] appears to report a salt form.

The 1D NOE spectrum provides evidence for the spatial proximity of the methylene linker to one aromatic proton whose resonance is split into a doublet of doublets (assigned to H‐5). The absence of an NOE between the methylene linker protons and H‐2 of the indole ring rules out *N* ^*1*^‐ACOM substitution. The corresponding HMBC spectrum (cf. Supporting Information S1: Figures S10–11) indicates the correlation of the methylene linker protons and a quaternary carbon of the aromatic indole core (assigned to C‐4), while no heteronuclear correlation between the methylene linker and the aminoethyl moiety of the tryptamine scaffold was detected. Taken together, these observations clearly point to *O*‐ACOM substitution. Further, the chemical shift of the methylene linker protons of **26** (δ(–O–C*H*_*2*_–O) = 5.95 ppm in methanol‐d_4_) agrees with the spectroscopic properties reported for **2** (δ(–O–C*H*_*2*_–O) = 5.92–5.96 ppm in CDCl_3_) [10] but clearly differs from *EE01/D(IV)* (δ(–X–C*H*_*2*_–O) = 5.60 ppm in methanol‐d_4_) [11]. As the chemical shift of the methylene linker protons for all *O*‐ACOM‐substituted compounds reported herein is between 5.86 and 5.97 ppm, irrespective of the solvent (DMSO‐d_6_, acetone‐d_6_, acetonitrile‐d_3_, methanol‐d_4_), this resonance may be regarded as a diagnostic signal. Clearly, **22**/**26** and *EE01/D(IV)* do not have the same connectivity.

*N* ^*1*^‐ACOM substitution is implausible for the previously reported compounds *EE01/D(IV)* and *EE02* according to the diagnostic methylene linker resonance [9, 10, 28], but the latter is in good agreement with *EE01/D(IV)* and *EE02* being quaternary ammonium salts in which the dimethylamino group has been alkylated (δ(–N–C*H*_*2*_–O) = 5.7–5.33 ppm in DMSO‐d_6_ or CDCl_3_) [29]. This assignment is further corroborated by the reported physical state of *EE01/D(IV)* and *EE02* (colorless solids) and the chemical shift of their dimethylamino group (*δ*(N(C*H*_*3*_)_2_) = 2.93 ppm in methanol‐d_4_). While being identical to the fumarate salt form **26** that bears a protonated dimethylamino group, the chemical shift differs significantly from the free base **22** (δ(N(C*H*_*3*_)_2_) = 2.26 ppm in acetonitrile‐d_3_) and a claimed acylated ACOM derivative, *EE03/D(XIX)* [11], which has been characterized in the same solvent (δ(N(C*H*_*3*_)_2_) = 2.35 ppm in methanol‐d_4_). Consequently, the aliphatic amino group of *EE01/D(IV)* and *EE02* appears to bear a positive charge, likely due to ACOM substitution. The mentioned ACOM derivatives *D(III)* and *EE03/D(XIX)* are, in contrast to their reported structures, most likely *N* ^*1*^‐ACOM derivatives as judged by their diagnostic methylene linker resonance (*δ*(–N–C*H*_*2*_–O) = 6.14–6.17 ppm in methanol‐d_4_) [9, 10, 28]. An overview of analytical characteristics and proposed molecular structures is given in Table 1.

This case study illustrates the difficulty of obtaining ACOM ethers of psilocin. The synthetic methodology is largely restricted to S_N_2 chemistry. Direct acyloxymethylation has higher tolerance for functional groups than chloromethylation but suffers from insufficient chemoselectivity. The formation of various regioisomers not only entails tedious purifications and minuscule yields but also bears the risk of inadvertently isolating an unwanted side product and confusing it with the targeted product. Our synthetic route mitigates this risk, enables higher yields, and provides access to novel ACOM ethers that are more readily cleaved than the sterically demanding pivaloyl congener **2**.

**Figure 5:**
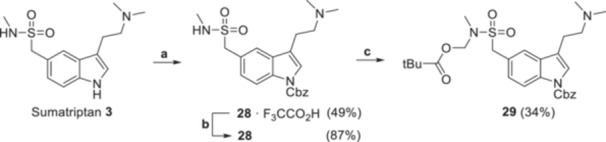
N^2^‐acyloxymethylation of sumatriptan via our synthetic strategy. **a.** 1.06 eq. 1‐Cbz‐imidazole, 0.71 eq DBU, MeCN, rt, 40 h; **b.** NaHCO_3_ (aq)/DCM extraction**. c.** DMF/THF, –50°C to rt 1) 2.12 eq NaH, 5 min; 2) 1.55 eq iodomethyl carboxylate, 3 h.

To test the versatility of our synthetic approach, we additionally targeted the unprecedented *N* ^*2*^‐acyloxymethyl derivatives of sumatriptan. As this drug suffers from several pharmacokinetic liabilities such as presystemic hepatic metabolism and low membrane permeability (logP 0.93) [9], *N* ^*2*^‐acyloxymethylation may be a useful tool for tailoring sumatriptan to the requirements of non‐oral dosage forms which avoid the first pass effect. To our delight, we obtained compound **29** without any optimization of the reaction conditions (Figure 5), which, to our knowledge, marks the first successful *N* ^*2*^‐acyloxymethylation of sumatriptan. Again, our structural assignment is based on spectroscopic analysis of the structures **28** and **29** (c.f. Supporting Information S1: Figures S12–13; Table S8). Comparison of the ^1^H NMR spectra of sumatriptan and **28** clearly shows the disappearance of the indole NH resonance upon installation of the Cbz group, along with a downfield shift of all aromatic proton resonances. Both observations suggest *N* ^*1*^‐substitution with the Cbz moiety, which is further corroborated by the HMBC spectrum of **29**. The latter documents mutual ^1^H,^13^C correlations between the *N*‐methyl and –NCH_2_O– groups, thus providing direct evidence for the reported connectivity.

This example demonstrates that the presented synthetic strategy is not limited to hydroxytryptamines but may be extended at least to selected triptans (e.g., sumatriptan, naratriptan, and avitriptan) and potentially beyond.

### 2.2 Pharmacology

We characterized the ACOM ethers **23**–**26** as well as the *N*‐carbamates **15** and **16** in vitro to evaluate their suitability as prodrugs (Table 2).

**Table 2:** In vitro pharmacokinetic and physicochemical characterization.

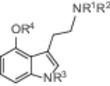					Passive membrane permeability		Half‐lives *t*_1/2_(min)			
								at 37°C in human		
Cpd.	R^1^	R^2^	R^3^	R^4^	P_e_(10^−6^cm/s)	R (%)	at 80°C in PBS^b^	Saliva^c^	10% plasma	100% plasma
**15**	Et	Me	Cbz	H	n.d.	n.d.	136 ± 4	n.d.	n.d.	> 5700^b^^**,**^ d
**16**	Me	Me	Cbz	H	n.d.	n.d.	184 ± 5	n.d.	n.d.	> 5700^b^^**,**^ d
**23** a	Et	Me	H	*t*BuCO_2_CH_2_	9.7 ± 0.6	2	534 ± 12	< 20	n.d.	> 240^b^
**24** a	Et	Me	H	nPrCO_2_CH_2_	8.0 ± 0.3	6	73.3 ± 1.2	< 10	4.2 ± 0.1^d^	n.d.
**25** a	Me	Me	H	tBuCO_2_CH_2_	9.1 ± 0.5	4	458 ± 23	< 20	n.d.	> 240^b^^**,**^ d
**26** a	Me	Me	H	nPrCO_2_CH_2_	8.1 ± 0.8^e^	7^e^	58.6 ± 3.5	< 10	3.5 ± 0.8^d^	0.48 ± 0.10
**References**										
**ASA**	Acetylsalicylic acid				n.d.	n.d	27.9 ± 0.3	> 60	n.d.	146 ± 19^f^
**30** a	4‐HO‐MET				11 ± 1	29	n.d.	n.d.	n.d.	n.d.

^a^ Fumarate salt.

^b^ No replicates.

^c^ Mean of three measurements performed with fresh samples of three individuals.

^d^ No clear distinction between zero and first order kinetics possible.

^e^ Mean of six replicates.

^f^ [ASA]_0_ = 560 µM; t_1/2_(lit.) = (130 ± 48) min [30].

As expected, all tested prodrugs have a high propensity for passive passage across an artificial membrane at pH 7.4 (≥ 8·10^−6^ cm/s). Interestingly, however, the parent compound **30** (tryptammonium fumarate salt form of **5** in 2:1 stoichiometry) had the highest membrane permeability. The loss of the intramolecular hydrogen bond [31] between the phenolic hydroxy group and the tertiary amino group upon *O*‐substitution appears to decrease the membrane permeability of the prodrugs in comparison to the parent drug. This effect is apparently not entirely compensated by the ACOM promoiety, which in itself is not completely apolar due to the acetal and ester oxygens. The intramolecular hydrogen bond may reduce the polarity of 4‐hydroxytryptamines by introducing a conformational restraint and thereby favoring conformations with a smaller polar surface area and a lower dipole moment and by decreasing hydrogen bonding to the aqueous solvent. In addition, the intramolecular hydrogen bond in 4‐hydroxytryptamines lowers the p*K*_a_ of the amine and therefore shifts the protonation equilibrium toward the neutral species. The higher proportion of the uncharged species in 4‐hydroxytryptamines compared with their *O*‐substituted congeners further increases the difference in passive membrane permeability [32]. It should be noted that the mass retention (R%) of **30** is remarkably high and the passive permeability reported here may therefore be inaccurate.

The *O*‐ACOM promoieties protect the notoriously labile 4‐hydroxytryptamines from oxidative degradation while maintaining a high membrane permeability and thereby provide benefits in comparison to the respective parent drug and phosphate ester prodrug (e.g., psilocybin).

**Figure 6:**
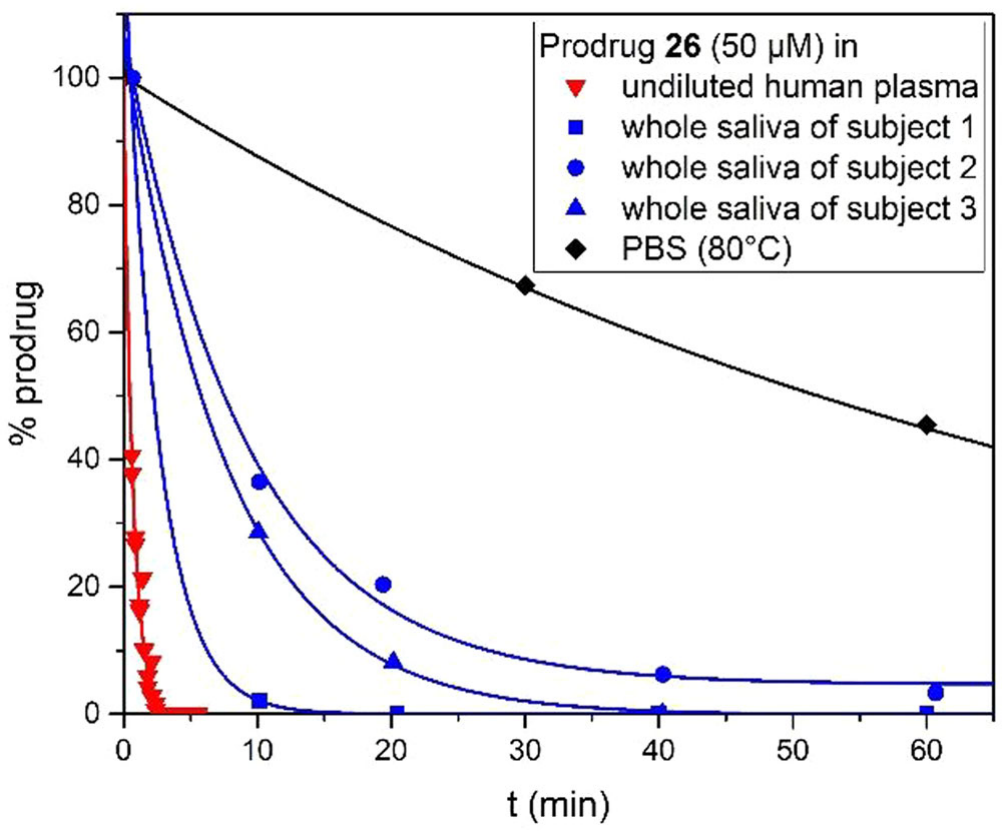
Stability profile of prodrug **26** in human media at 37°C and PBS at 80°C.

As a measure of the drug release rate in the systemic blood circulation, we determined the half‐lives of **15**, **16**, and **23**–**26** in pooled human plasma. Neither the *N*‐carbamates **15** and **16** nor the sterically hindered pivaloyloxymethyl ethers **23** and **25** showed significant drug release in undiluted human plasma during the observation period. In contrast, half‐lives below 5 min were observed for the ACOM ethers **24** and **26**, even in diluted human plasma. This illustrates that the drug release kinetics in human plasma are highly dependent upon the chosen acyl residue, which allows for tailoring the prodrug's pharmacokinetic profile to the therapeutic requirements. To assess the contribution of nonspecific hydrolysis to the observed kinetics, we incubated the prodrugs at the same concentration in the buffer used to dilute the plasma samples. Even at 80°C, **24** and **26** were significantly more stable in buffer than in plasma (Figure 6), which supports the assumption of enzymatic prodrug cleavage.

**Figure 7:**
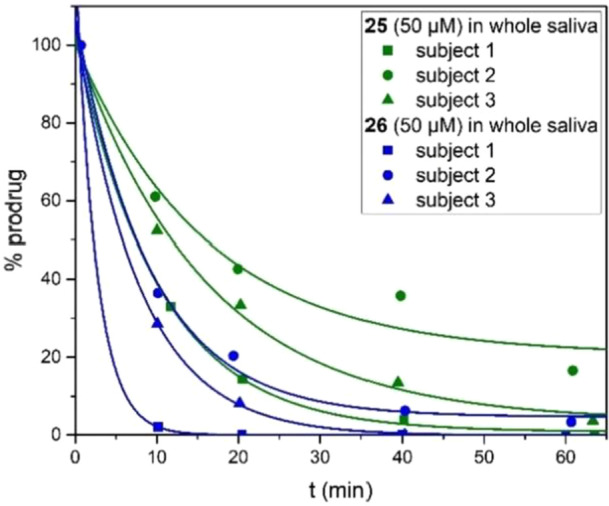
Interindividual variability of the stability in human whole saliva at 37°C.

Finally, we studied the stability of the compounds in human saliva. Considering the large interindividual variability of the salivary esterase activity [33], we determined the half‐life of every compound in samples of resting saliva obtained from three subjects and grouped the mean half‐lives into four categories (< 10 min, 10–20 min, 20–60 min, and > 60 min). In line with the literature, the stability of a given compound in human saliva varied considerably between subjects (Figure 7).

Despite the significant interindividual variability, it is evident that the assayed ACOM ethers are extremely labile in human saliva, irrespective of their steric hindrance. Taking into account that the half‐lives were determined in resting saliva (without prior paraffin chewing to stimulate the salivary glands) and the stability in vivo may thus be even lower than observed in vitro [34], we consider compounds **23**–**26** and related ACOM ethers of 4‐hydroxytryptamines to be unsuitable for sublingual or buccal dosage forms.

The unanticipated rapid cleavage of all assayed ACOM ethers in human saliva appears to reflect the presence of enzymes that are absent in human plasma. The marginal difference in the kinetics of ACOM ethers bearing *tert*‐butyl and *n*‐propyl residues provides information on the predominantly operative hydrolysis mechanism. An enzymatic hydrolysis mechanism where the carboxylate moiety acts as a leaving group is more plausible than a hydrolysis mechanism via tetrahedral intermediates since the latter would be significantly affected by the acyl residue's steric demand. Amylases may be involved in ACOM ether cleavage, since they are relatively abundant in human saliva [35].

Of note, the lack of stability in saliva does not preclude oral dosing if the formulation of peroral dosage forms provides protection from saliva and gastric fluids, for example, by an appropriate coating. In fact, Clark et al. have reported that **2** (corresponding to fumarate **25**) acts as a psilocin prodrug in rats upon peroral dosing [10b], which may be considered as a proof of principle for ACOM ether prodrugs of psilocin. The detected systemic psilocin level upon administration of **2** was, however, relatively low. This may be ascribed to the high chemical and plasma stability which we observed in vitro for **25**. Regarding the more readily cleaved ACOM ether **26**, we would expect a slightly faster onset and a higher systemic psilocin level when administered orally.

## 3 Conclusion

We have developed a novel route for the acyloxymethylation of psilocin and related tryptamines addressing the chemoselectivity issues arising from the presence of multiple (pro)nucleophilic groups in the drug molecules. As exemplified by the *N* ^*2*^‐acyloxymethylation of sumatriptan (sulfonamide pK_a_ ~ 17.5 in DMSO [36]), our strategy may be extended toward compounds where the indole NH is the only functional group with a p*K*_a_ value of 17.5–23 (in DMSO) [22]. Given the abundance of indole derivatives in medicinal chemistry, our methodology should grant synthetic access to a variety of novel ACOM prodrugs.

In addition, we have demonstrated in vitro that ACOM ethers of 4‐hydroxytryptamines, such as the novel *n*‐butyryl congeners **24** and **26**, are viable prodrugs. The pharmacokinetic profile of the ACOM ether prodrugs primarily depends on the nature of the promoiety's acyl residue and, to a lesser extent, on the substituents of the aliphatic amino group, which qualifies 4‐hydroxy‐*N*‐ethyl‐*N*‐methyltryptamine **5** as a model for the more strictly regulated psilocin **1**. With regard to the further exploration of the structure–activity relationships of aliphatic ACOM ether prodrugs of 4‐hydroxytryptamines, the in vitro data presented herein may serve as an estimate of the variation range of their pharmacokinetic characteristics and should assist with tailoring ACOM ether prodrugs to the requirements of a psychedelic treatment session. While the unanticipated lability in human saliva likely precludes sublingual or buccal application, other routes of administration such as peroral [17], intravenous, nasal, or transdermal [37] merit further evaluation.

## 4 Experimental

### 4.1 Chemistry

Exemplary experimental procedures are given below. Additional experimental details and procedures are provided as Supporting Information. The InChI codes of the investigated compounds, together with some biological activity data, are provided as Supporting Information.

#### 4.1.1 Synthesis of 1‐Benzyloxycarbonyl‐4‐Hydroxy‐*N*,*N*‐Dimethyltryptamine (16)


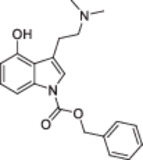


A flame‐dried Schlenk flask was charged with 4‐hydroxy‐*N*,*N*‐dimethyltryptamine **1** (1.36 g, 6.66 mmol, 1.00 eq). Benzyl 1*H*‐imidazole‐1‐carboxylate **S1** (1.48 g, 7.32 mmol, 1.10 eq) dissolved in anhydrous acetonitrile (20 mL) was added, followed by 1,8‐diazabicyclo[5.4.0]undec‐7‐ene (497 µL, 507 mg, 3.33 mmol, 0.50 eq). The resulting brown solution was stirred for 26 h at ambient temperature. Afterward, the reaction mixture was concentrated by rotary evaporation, quenched by the addition of 0.5 M aqueous NaOH (35 mL), and the mixture was extracted with dichloromethane (3 × 200 mL). The pooled organic layers were dried over Na_2_SO_4_ and concentrated in vacuo. Purification by column chromatography (EtOAc/NEt_3_ 99/1 (v/v), SiO_2_ 50 g) afforded **16** (1.35 g, 3.99 mmol, 60%) as a yellowish oil which crystallized upon standing.

^1^H NMR (500 MHz, CDCl_3_): *δ* = 13.35 (br s, 1H, O*H*), 7.73 (br d, *J* = 5.1 Hz, 1H, Ar*H*), 7.44–7.52 (m, 2H, Ar*H*), 7.33–7.44 (m, 3H, Ar*H*), 7.28 (s, 1H, Ar*H*), 7.19 (t, *J* = 8.1 Hz, 1H, Ar*H*), 6.74 (dd, *J* = 7.9 Hz, *J‘* = 0.6 Hz, 1H, Ar*H*), 5.42 (s, 2H, OC*H*_*2*_), 2.84–2.92 (m, 2H, ArC*H*_*2*_CH_2_), 2.66–2.74 (m, 2H, ArCH_2_C*H*_*2*_), 2.37 (s, 6H, N(C*H*_*3*_)_2_) ppm. ^13^C{^1^H} NMR (126 MHz, CDCl_3_): *δ* = 152.2 (C_q_, 1 C, Ar), 150.9 (C_q_, 1 C, C═O), 137.9 (C_q_, 1 C, Ar), 135.4 (C_q_, 1 C, Ar), 128.8 (CH, 2 C, Ar), 128.7 (CH, 1 C, Ar), 128.5 (CH, 2 C, Ar), 126.2 (CH,1 C, Ar), 121.8 (CH,1 C, Ar), 120.0 (C_q_, 1 C, Ar), 119.7 (C_q_, 1 C, Ar), 111.4 (CH, 1 C, Ar), 106.6 (CH, 1 C, Ar), 68.5 (CH_2_, 1 C, O*C*H_2_), 61.0 (CH_2_, 1 C, ArCH_2_ *C*H_2_), 45.4 (CH_3_, 2C, N(*C*H_3_)_2_), 25.2 (CH_2_, 1 C, Ar*C*H_2_CH_2_) ppm. HR MS (ESI^+^): *m/z* [M+Na]^+^ calcd. for [C_20_H_22_N_2_NaO_3_]^+^: 361.1523, found 361.1512; [M+H]^+^ calcd. for [C_20_H_23_N_2_O_3_]^+^: 339.1703, found 339.1701.

#### 4.1.2 Synthesis of 1‐Benzyloxycarbonyl‐4‐*n*‐Butanoyloxymethyloxy‐*N*,*N*‐Dimethyltryptammonium Trifluoroacetate (20)


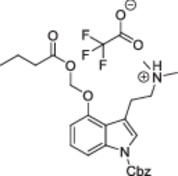


A solution of **16** (400 mg, 1.18 mmol, 1.00 eq) in anhydrous DMF (5.6 mL) was treated with NaH (60% suspension in mineral oil, 70.9 mg, 1.77 mmol, 1.50 eq) at –30°C to –45°C. After stirring for 30 min at this temperature, iodomethyl butyrate **S3** (404 mg, 1.77 mmol, 1.50 eq) as a solution in anhydrous THF (5.6 mL) was slowly added. The reaction mixture was allowed to slowly warm to room temperature and stirred for a further 5 h. Subsequently, the reaction mixture was poured into aq. 1 N NaOH (5.4 mL) and extracted with ethyl acetate (3 × 20 mL). The organic layer was dried over Na_2_SO_4_, concentrated, dissolved in acetonitrile (+0.1% TFA), and purified by means of reversed‐phase chromatography on a preparative HPLC instrument (Supporting Information S1: Table S1, gradient I). Evaporation of volatiles followed by lyophilization afforded **20** (96.8 mg, 171 µmol, 14%) as a colorless oil.

^1^H NMR (500 MHz, CD_3_CN): *δ* = 10.74 (br s, 1H, N*H*), 7.82 (d, *J* = 8.3 Hz, 1H, Ar*H*), 7.50 (d, *J* = 7.1 Hz, 2H, Ar*H*), 7.46 (s, 1H, Ar*H*), 7.35–7.45 (m, 3H, Ar*H*), 7.27 (t, *J* = 8.3 Hz, 1H, Ar*H*), 6.95 (d, *J* = 8.1 Hz, 1H, Ar*H*), 5.90 (s, 2H, OC*H*_*2*_O), 5.41 (s, 2H, OC*H*_*2*_Ph), 3.24–3.35 (m, 2H, ArCH_2_C*H*_*2*_), 3.10–3.22 (m, 2H, ArC*H*_*2*_CH_2_), 2.86 (s, 6H, N(*C*H_3_)_2_), 2.32 (t, *J* = 7.3 Hz, 2H, (C═O)C*H*_*2*_), 1.57 (sxt, *J* = 7.4 Hz, 2H, CH_3_C*H*_*2*_), 0.85 (t, *J* = 7.5 Hz, 3H, C*H*_*3*_CH_2_) ppm. ^13^C{^1^H} NMR (126 MHz, CD_3_CN): *δ* = 173.7 (C_q_, 1 C, CH_2_ *C*O_2_), 161.2 (C_q_, q, *J*_*C–F*_ = 35.1 Hz, 1 C, TFA), 151.3 (C_q_, 1 C, N*C*O_2_), 151.2 (C_q_, 1 C, Ar), 138.4 (C_q_, 1 C, Ar), 136.5 (C_q_, 1 C, Ar), 129.6 (CH, 2 C, Ar), 129.6 (CH, 1 C, Ar), 129.3 (CH, 2 C, Ar), 126.8 (CH, 1 C, Ar), 124.6 (CH, 1 C, Ar), 120.4 (C_q_, 1 C, Ar), 117.6 (C_q_, q, *J*_*C–F*_ = 292.5 Hz, 1 C, TFA), 116.3 (C_q_, 1 C, Ar), 110.6 (CH, 1 C, Ar), 107.3 (CH, 1 C, Ar), 85.4 (CH_2_, 1 C, O*C*H_2_O), 69.6 (CH_2_, 1 C, O*C*H_2_Ph), 58.7 (CH_2_, 1 C, ArCH_2_ *C*H_2_), 43.3 (CH_3_, 2C, N(*C*H_3_)_2_), 36.4 (CH_2_, 1 C, (C═O)*C*H_2_), 22.7 (CH_2_, 1 C, Ar*C*H_2_CH_2_), 18.8 (CH_2_, 1 C, CH_3_ *C*H_2_), 13.7 (CH_3_, 1 C, *C*H_3_CH_2_) ppm. ^19^F NMR (282 MHz, CD_3_CN): *δ* = –76.1 (s, 3 F, TFA) ppm. HR MS (ESI^+^): *m/z* [M+H]^+^ calcd. for [C_25_H_31_N_2_O_5_]^+^: 439.2227, found 439.2224.

#### 4.1.3 Synthesis of 4‐*n*‐Butanoyloxymethyloxy‐*N*,*N*‐Dimethyltryptammonium Fumarate (26)


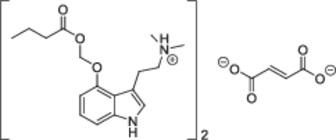


A 50‐mL two‐necked flask was charged with 10% Pd/C (26.2 mg, 24.7 µmol, 0.15 eq). After three evacuation‐backfill cycles with nitrogen, the flask was filled with hydrogen gas and equipped with a hydrogen‐containing balloon. Then, a solution of **20** (90.1 mg, 163 µmol, 1.00 eq) in EtOAc (13 mL) was added via syringe, and the reaction mixture was stirred at room temperature for 6 h. Subsequently, the reaction mixture was filtered through a pad of cotton. The filter cake was rinsed with EtOAc (3 × 2 mL) and concentrated by rotary evaporation. The crude was then dissolved in ACN/H_2_O (1:1, +0.1% TFA) and purified by means of reversed‐phase chromatography on a preparative HPLC instrument (gradient II). The product fractions were concentrated by rotary evaporation, set to pH 10–11 using 1 N NaOH (2.9 mL), and extracted with dichloromethane (3 × 45 mL). Drying over Na_2_SO_4_ followed by rotary evaporation afforded 4‐*n*‐butanoyloxymethyloxy‐*N*,*N*‐dimethyltryptamine **22** (40.4 mg, 133 µmol, 81%) as a yellowish oil, which was used in the next step without further purification.

^1^H NMR (500 MHz, CD_3_CN): *δ* = 9.32 (br s, 1H, N*H*), 7.04–7.09 (m, 1H, Ar*H*), 6.98–7.04 (m, 1H, Ar*H*), 6.96 (d, *J* = 1.7 Hz, 1H, Ar*H*), 6.66 (d, *J* = 7.6 Hz, 1H, Ar*H*), 5.86 (s, 2H, OC*H*_*2*_O), 2.92–3.01 (m, 2H, ArC*H*_*2*_CH_2_), 2.50–2.59 (m, 2H, ArCH_2_C*H*_*2*_), 2.32 (t, *J* = 7.3 Hz, 2H, (C═O)C*H*_*2*_), 2.26 (s, 6H, N(C*H*_*3*_)_2_), 1.60 (sxt, *J* = 7.4 Hz, 2H, (C═O)CH_2_C*H*_*2*_), 0.89 (t, *J* = 7.4 Hz, 3H, C*H*_*3*_(CH_2_)_2_) ppm. ^13^C{^1^H} NMR (126 MHz, CD_3_CN): *δ* = 173.4 (C_q_, 1 C, C═O), 152.2 (C_q_, 1 C, Ar), 139.5 (C_q_, 1 C, Ar), 123.0 (CH, 1 C, Ar), 122.9 (CH, 1 C, Ar), 118.8 (C_q_, 1 C, Ar), 114.4 (C_q_, 1 C, Ar), 107.4 (CH, 1 C, Ar), 103.3 (CH, 1 C, Ar), 86.3 (CH_2_, 1 C, O*C*H_2_O), 62.2 (CH_2_, 1 C, ArCH_2_ *C*H_2_), 45.6 (CH_3_, 2C, N(*C*H_3_)_2_), 36.6 (CH_2_, 1 C, (C═O)*C*H_2_), 25.5 (CH_2_, 1 C, Ar*C*H_2_CH_2_), 18.9 (CH_2_, 1 C, (C═O)CH_2_ *C*H_2_), 13.7 (CH_3_, 1 C, *C*H_3_(CH_2_)_2_) ppm.

To a solution of fumaric acid (6.65 mg, 56.7 µmol, 0.43 eq) in acetone (1.4 mL), **22** (40.4 mg, 133 µmol, 1.00 eq) dissolved in acetone (0.8 mL) was added, and the obtained mixture was thoroughly vortexed. After standing for 6 h at 5°C, the suspension was centrifuged (3500 RCF, 5 min) and decanted. The residue was washed with cold (5°C) acetone (2 × 0.3 mL) and lyophilized, which afforded **26** (33.2 mg, 91.6 µmol, 69%) as a colorless solid.

^1^H NMR (CD_3_OD, 500 MHz): *δ* = 7.08 (s, 1H, Ar*H*), 7.01–7.07 (m, 2H, Ar*H*), 6.72 (dd, *J* = 6.1 Hz, *J’* = 2.3 Hz, 1H, Ar*H*), 6.68 (s, 1H, fumarate), 5.95 (s, 2H, OC*H*_*2*_O), 3.33–3.39 (m, 2H, ArCH_2_C*H*_*2*_), 3.19–3.26 (m, 2H, ArC*H*_*2*_CH_2_), 2.93 (s, 6H, N(C*H*_*3*_)_2_), 2.36 (t, *J* = 7.3 Hz, 2H, (C═O)C*H*_*2*_), 1.62 (sxt, *J* = 7.3 Hz, 2H, (C═O)CH_2_C*H*_*2*_), 0.88 (t, *J* = 7.4 Hz, 3H, C*H*_*3*_(CH_2_)_2_) ppm. ^13^C{^1^H} NMR (126 MHz, CD_3_OD): *δ* = 174.7 (C_q_, 1 C, CH_2_ *C*O_2_), 173.2 (C_q_, 1 C, fumarate), 151.5 (C_q_, 1 C, Ar), 140.5 (C_q_, 1 C, Ar), 136.7 (CH, 1 C, fumarate), 124.2 (CH, 1 C, Ar), 123.4 (CH, 1 C, Ar), 118.4 (C_q_,1 C, Ar), 109.8 (C_q_, 1 C, Ar), 107.6 (CH, 1 C, Ar), 102.6 (CH, 1 C, Ar), 85.5 (CH_2_, 1 C, O*C*H_2_O), 60.7 (CH_2_, 1 C, ArCH_2_ *C*H_2_), 43.7 (CH_3_, 2C, N(*C*H_3_)_2_), 36.9 (CH_2_, 1 C, (C═O)C*H*_*2*_), 23.7 (CH_2_, 1 C, ArC*H*_*2*_CH_2_), 19.3 (CH_2_, 1 C, (C═O)CH_2_ *C*H_2_), 13.8 (CH_3_, 1 C, C*H*_*3*_(CH_2_)_2_) ppm. ^1^H NMR (300 MHz, CD_3_CN): *δ* = 9.27 (br s, 1H, N_ind_ *H*), 6.98–7.12 (m, 3H, Ar*H*), 6.69 (dd, *J* = 7.2 Hz, *J’* = 1.3 Hz, 1H, Ar*H*), 6.61 (s, 1H, fumarate), 5.90 (s, 2H, OC*H*_*2*_O), 3.06–3.18 (m, 2H, ArCH_2_C*H*_*2*_), 2.97–3.06 (m, 2H, ArC*H*_*2*_CH_2_), 2.63 (s, 6H, N(C*H*_*3*_)_2_), 2.33 (t, *J* = 7.3 Hz, 2H, (C═O)C*H*_*2*_), 1.59 (sxt, *J* = 7.4 Hz, 2H, (C═O)CH_2_C*H*_*2*_), 0.87 (t, *J* = 7.5 Hz, 3H, C*H*_*3*_(CH_2_)_2_) ppm. ^1^H NMR (500 MHz, (CD_3_)_2_SO): *δ* = 10.92 (br s, 1H, N_ind_ *H*), 7.07 (d, *J* = 1.7 Hz, 1H, Ar*H*), 7.01–7.05 (m, 1H, Ar*H*), 6.95–7.01 (m, 1H, Ar*H*), 6.63 (d, *J* = 7.6 Hz, 1H, Ar*H*), 6.50 (s, 1H, fumarate), 5.89 (s, 2H, OC*H*_*2*_O), 2.92–3.03 (m, 2H, ArC*H*_*2*_CH_2_), 2.73–2.86 (m, 2H, ArCH_2_C*H*_*2*_), 2.46 (s, 6H, N(C*H*_*3*_)_2_), 2.34 (t, *J* = 7.3 Hz, 2H, (C═O)C*H*_*2*_), 1.54 (sxt, *J* = 7.3 Hz, 2H, (C═O)CH_2_C*H*_*2*_), 0.84 (t, *J* = 7.4 Hz, 3H, C*H*_*3*_(CH_2_)_2_) ppm. ^13^C{^1^H} NMR (126 MHz, (CD_3_)_2_SO): *δ* = 172.1 (C_q_, 1 C, CH_2_ *C*O_2_), 167.5 (C_q_, 1 C, fumarate), 150.4 (C_q_, 1 C, Ar), 138.3 (C_q_, 1 C, Ar), 134.9 (CH, 1 C, fumarate), 122.6 (CH, 1 C, Ar), 121.6 (CH, 1 C, Ar), 117.2 (C_q_, 1 C, Ar), 110.8 (C_q_, 1 C, Ar), 106.4 (CH, 1 C, Ar), 101.6 (CH, 1 C, Ar), 84.8 (CH_2_, 1 C, O*C*H_2_O), 59.7 (CH_2_, 1 C, ArCH_2_ *C*H_2_), 43.6 (CH_3_, 2C, N(*C*H_3_)_2_), 35.3 (CH_2_, 1 C, (C═O)*C*H_2_), 23.0 (CH_2_, 1 C, Ar*C*H_2_CH_2_), 17.7 (CH_2_, 1 C, (C═O)CH_2_ *C*H_2_), 13.2 (CH_3_, 1 C, *C*H_3_(CH_2_)_2_) ppm. HR MS (ESI^+^): *m/z* [M+Na]^+^ calcd. for [C_17_H_24_N_2_NaO_3_]^+^: 327.1679, found 327.1667; [M+H]^+^ calcd. for [C_17_H_25_N_2_O_3_]^+^: 305.1860, found 305.1852.

### 4.2 Half‐Lives in PBS, Human Saliva, and Human Plasma

The kinetic studies were carried out by following the loss of substrate under the indicated conditions using HPLC‐UV. Pooled human plasma was obtained commercially (Biowest, France), while human saliva was freshly collected with the written informed consent of the saliva donors. In a typical run, the reaction was initiated by adding the prewarmed medium to a prewarmed aliquot of a 5 mM stock solution of the analyte in DMSO, resulting in a final concentration of 5·10^−5^ M. A volume of 100 µL samples of the reaction mixture were taken at suitable intervals, and the reaction was either stopped by cooling to –20°C (PBS) or by protein precipitation using 2:1 (v/v) acetonitrile (saliva, plasma) [38]. Precipitates were removed by centrifugation and decantation prior to HPLC analysis. Pseudo‐first‐order rate constants for the observed degradation were determined from the slope of linear plots of ln(peak area) versus time. More detailed descriptions are provided in the Supporting Information.

## Conflicts of Interest

Heidelberg University has filed a patent application (EP24154403.0) covering the 4‐hydroxytryptamine derivatives presented herein. The authors are named as inventors on this patent application.

## Supporting information

ArchPharm SupplMat InChI.

## Data Availability

Additional NMR data are available upon request from the authors.
