# Supplementary material for: Efficient Acyloxymethylation of Psilocin and Other Tryptamines Yielding ACOM Prodrugs for Psychedelic‐Assisted Therapy
Source: Arch Pharm (Weinheim). 2025 Jul 23;358(7):e70022. doi: 10.1002/ardp.70022 (PMC12287680; doi:10.1002/ardp.70022)
Supplement: Supplementary file 1 — ArchPharm SupplMat InChI. [file ARDP-358-e70022-s001.doc]

**Supplemental Material: Novel Compounds and Biological Screening Results**

**Efficient Acyloxymethylation of Psilocin and Other Tryptamines Yielding ACOM Prodrugs for Psychedelic-Assisted Therapy**

Judith Stirn, Christian D. Klein*

Medicinal Chemistry, Institute of Pharmacy and Molecular Biotechnology IPMB, Heidelberg University, Im Neuenheimer Feld 364, D-69120 Heidelberg, Germany

*Email: c.klein@uni-heidelberg.de

ORCIDs:

Judith Stirn orcid.org/0009-0007-9105-3894

Christian D. Klein orcid.org/0000-0003-3522-9182

| **Compound No.**  **Trivial name** | **InChI** | **half-lives t_1/2_ [min] in human plasma (v/v) at 37°C, [prodrug]_0_ =50 µM^a^** |
| --- | --- | --- |
| **1** | InChI=1S/C12H16N2O/c1-14(2)7-6-9-8-13-10-4-3-5-11(15)12(9)10/h3-5,8,13,15H,6-7H2,1-2H3 | Not determined |
| **2** | InChI=1S/C18H26N2O3/c1-18(2,3)17(21)23-12-22-15-8-6-7-14-16(15)13(11-19-14)9-10-20(4)5/h6-8,11,19H,9-10,12H2,1-5H3 | Not determined |
| **3** | Not isolated | Not determined |
| **4** | Not isolated | Not determined |
| **5** | InChI=1S/C13H18N2O/c1-3-15(2)8-7-10-9-14-11-5-4-6-12(16)13(10)11/h4-6,9,14,16H,3,7-8H2,1-2H3 | Not determined |
| **6** | Not isolated | Not determined |
| **7** | Not isolated | Not determined |
| **8** | InChI=1S/C19H28N2O3/c1-6-21(5)11-10-14-12-20-15-8-7-9-16(17(14)15)23-13-24-18(22)19(2,3)4/h7-9,12,20H,6,10-11,13H2,1-5H3 | Not determined |
| **9** | InChI=1S/C15H16N2O4/c1-4-17(3)15(20)14(19)10-8-16-11-6-5-7-12(13(10)11)21-9(2)18/h5-8,16H,4H2,1-3H3 | Not determined |
| **10** | InChI=1S/C24H36N2O4Si/c1-10-25(9)24(29)23(28)19-14-26(31(15(2)3,16(4)5)17(6)7)20-12-11-13-21(22(19)20)30-18(8)27/h11-17H,10H2,1-9H3 | Not determined |
| **11** | Not isolated | Not determined |
| **12** | InChI=1S/C18H26N2O3/c1-6-20(5)11-10-13-12-19-14-8-7-9-15(16(13)14)22-17(21)23-18(2,3)4/h7-9,12,19H,6,10-11H2,1-5H3 | Not determined |
| **13** | InChI=1S/C23H34N2O5/c1-9-24(8)14-13-16-15-25(20(26)29-22(2,3)4)17-11-10-12-18(19(16)17)28-21(27)30-23(5,6)7/h10-12,15H,9,13-14H2,1-8H3 | Not determined |
| **14** | InChI=1S/C18H26N2O3/c1-6-19(5)11-10-13-12-20(17(22)23-18(2,3)4)14-8-7-9-15(21)16(13)14/h7-9,12,21H,6,10-11H2,1-5H3 | Not determined |
| **15** | InChI=1S/C21H24N2O3/c1-3-22(2)13-12-17-14-23(18-10-7-11-19(24)20(17)18)21(25)26-15-16-8-5-4-6-9-16/h4-11,14,24H,3,12-13,15H2,1-2H3 | > 5700 (100%) |
| **16** | InChI=1S/C20H22N2O3/c1-21(2)12-11-16-13-22(17-9-6-10-18(23)19(16)17)20(24)25-14-15-7-4-3-5-8-15/h3-10,13,23H,11-12,14H2,1-2H3 | > 5700 (100%) |
| **17** | InChI=1S/C27H34N2O5.C2HF3O2/c1-6-28(5)16-15-21-17-29(26(31)32-18-20-11-8-7-9-12-20)22-13-10-14-23(24(21)22)33-19-34-25(30)27(2,3)4;3-2(4,5)1(6)7/h7-14,17H,6,15-16,18-19H2,1-5H3;(H,6,7) | Not determined |
| **18** | InChI=1S/C26H32N2O5.C2HF3O2/c1-4-10-24(29)33-19-32-23-14-9-13-22-25(23)21(15-16-27(3)5-2)17-28(22)26(30)31-18-20-11-7-6-8-12-20;3-2(4,5)1(6)7/h6-9,11-14,17H,4-5,10,15-16,18-19H2,1-3H3;(H,6,7) | Not determined |
| **19** | InChI=1S/C26H32N2O5.C2HF3O2/c1-26(2,3)24(29)33-18-32-22-13-9-12-21-23(22)20(14-15-27(4)5)16-28(21)25(30)31-17-19-10-7-6-8-11-19;3-2(4,5)1(6)7/h6-13,16H,14-15,17-18H2,1-5H3;(H,6,7) | Not determined |
| **20** | InChI=1S/C25H30N2O5.C2HF3O2/c1-4-9-23(28)32-18-31-22-13-8-12-21-24(22)20(14-15-26(2)3)16-27(21)25(29)30-17-19-10-6-5-7-11-19;3-2(4,5)1(6)7/h5-8,10-13,16H,4,9,14-15,17-18H2,1-3H3;(H,6,7) | Not determined |
| **21** | InChI=1S/C18H26N2O3/c1-4-7-17(21)23-13-22-16-9-6-8-15-18(16)14(12-19-15)10-11-20(3)5-2/h6,8-9,12,19H,4-5,7,10-11,13H2,1-3H3 | Not determined |
| **22** | InChI=1S/C17H24N2O3/c1-4-6-16(20)22-12-21-15-8-5-7-14-17(15)13(11-18-14)9-10-19(2)3/h5,7-8,11,18H,4,6,9-10,12H2,1-3H3 | Not determined |
| **23** | InChI=1S/2C19H28N2O3.C4H4O4/c2*1-6-21(5)11-10-14-12-20-15-8-7-9-16(17(14)15)23-13-24-18(22)19(2,3)4;5-3(6)1-2-4(7)8/h2*7-9,12,20H,6,10-11,13H2,1-5H3;1-2H,(H,5,6)(H,7,8)/b;;2-1+ | > 240 (100%) |
| **24** | InChI=1S/2C18H26N2O3.C4H4O4/c2*1-4-7-17(21)23-13-22-16-9-6-8-15-18(16)14(12-19-15)10-11-20(3)5-2;5-3(6)1-2-4(7)8/h2*6,8-9,12,19H,4-5,7,10-11,13H2,1-3H3;1-2H,(H,5,6)(H,7,8)/b;;2-1+ | 4.2 ± 0.1 (10%)^b^ |
| **25** | InChI=1S/2C18H26N2O3.C4H4O4/c2*1-18(2,3)17(21)23-12-22-15-8-6-7-14-16(15)13(11-19-14)9-10-20(4)5;5-3(6)1-2-4(7)8/h2*6-8,11,19H,9-10,12H2,1-5H3;1-2H,(H,5,6)(H,7,8)/b;;2-1+ | > 240 (100%)^b^ |
| **26** | InChI=1S/2C17H24N2O3.C4H4O4/c2*1-4-6-16(20)22-12-21-15-8-5-7-14-17(15)13(11-18-14)9-10-19(2)3;5-3(6)1-2-4(7)8/h2*5,7-8,11,18H,4,6,9-10,12H2,1-3H3;1-2H,(H,5,6)(H,7,8)/b;;2-1+ | 0.48 ± 0.10 (100%)  3.5 ± 0.8 (10%)^b^ |
| **27** | InChI=1S/C19H28N2O3.CH4O3S/c1-6-21(5)11-10-14-12-20-15-8-7-9-16(17(14)15)23-13-24-18(22)19(2,3)4;1-5(2,3)4/h7-9,12,20H,6,10-11,13H2,1-5H3;1H3,(H,2,3,4) | Not determined |
| **28** | InChI=1S/C14H21N3O2S/c1-15-20(18,19)10-11-4-5-14-13(8-11)12(9-16-14)6-7-17(2)3/h4-5,8-9,15-16H,6-7,10H2,1-3H3 | Not determined |
| **29** | InChI=1S/C22H27N3O4S/c1-23-30(27,28)16-18-9-10-21-20(13-18)19(11-12-24(2)3)14-25(21)22(26)29-15-17-7-5-4-6-8-17/h4-10,13-14,23H,11-12,15-16H2,1-3H3 | Not determined |
| **30** | InChI=1S/C28H37N3O6S/c1-28(2,3)26(32)37-20-30(6)38(34,35)19-22-12-13-25-24(16-22)23(14-15-29(4)5)17-31(25)27(33)36-18-21-10-8-7-9-11-21/h7-13,16-17H,14-15,18-20H2,1-6H3 | Not determined |
| **31** | InChI=1S/2C13H18N2O.C4H4O4/c2*1-3-15(2)8-7-10-9-14-11-5-4-6-12(16)13(10)11;5-3(6)1-2-4(7)8/h2*4-6,9,14,16H,3,7-8H2,1-2H3;1-2H,(H,5,6)(H,7,8)/b;;2-1+ | Not determined |
| **ASA^c^** | InChI=1S/C9H8O4/c1-6(10)13-8-5-3-2-4-7(8)9(11)12/h2-5H,1H3,(H,11,12) | 146 ± 19 (100%) |
| **S1** | InChI=1S/C11H10N2O2/c14-11(13-7-6-12-9-13)15-8-10-4-2-1-3-5-10/h1-7,9H,8H2 | Not determined |
| **S2** | InChI=1S/C6H11IO2/c1-6(2,3)5(8)9-4-7/h4H2,1-3H3 | Not determined |
| **S3** | InChI=1S/C5H9IO2/c1-2-3-5(7)8-4-6/h2-4H2,1H3 | Not determined |

^a^ The release kinetics of the prodrugs were evaluated *in vitro* in analogy to the precedent literature.^[1]^ As ZnSO_4_ was found to affect the analytes’ signal intensity presumably by complexation, acetonitrile (2:1) was used as protein precipitation agent.^[2]^ Pooled human blood plasma (Biowest, France) and sterile Dulbecco’s phosphate buffered saline (PBS) pH 7.3 (Sigma-Aldrich, Germany) were used for all experiments. In short, prewarmed (37°C) human plasma and prewarmed (37°C) prodrug (5 mM stock in DMSO) were gently mixed, which afforded a 50 µM solution of the prodrug in human plasma. Incubation was continued at 37°C, and 100 µL samples of the mixture were taken at appropriate time intervals. The samples were immediately quenched with acetonitrile (2:1), centrifuged, decanted, and analyzed via HPLC-UV. Half-lives were determined assuming first-order kinetics; specific values are the mean of triplicates ± standard deviation. For more details, please refer to the SI.

^b^No clear distinction between zero and first order kinetics possible.

^c^ [ASA]_0_ = 560 µM; t_1/2_ (lit.) =(130 ± 48) min.^[3]^

[1] L. L. Christrup, C. B. Christensen, G. J. Friis, A. Jorgensen, *Int. J. Pharm.* **1997**, *154*, 157, DOI: 10.1016/S0378-5173(97)00128-2.

[2] C. Polson, P. Sarkar, B. Incledon, V. Raguvaran, R. Grant, *J. Chromatogr. B* **2003**, *785*, 263, DOI: 10.1016/S1570-0232(02)00914-5.

[3] L. Harthon, M. Hedströom, *Acta Pharmacol. Toxicol.* **1971**, *29*, 155, DOI: 10.1111/j.1600-0773.1971.tb00602.x.
